# *Haemophilus influenzae* OxyR: Characterization of Its Regulation, Regulon and Role in Fitness

**DOI:** 10.1371/journal.pone.0050588

**Published:** 2012-11-30

**Authors:** Paul W. Whitby, Daniel J. Morton, Timothy M. VanWagoner, Thomas W. Seale, Brett K. Cole, Huda J. Mussa, Phillip A. McGhee, Chee Yoon S. Bauer, Jennifer M. Springer, Terrence L. Stull

**Affiliations:** 1 Department of Pediatrics, University of Oklahoma Health Sciences Center, Oklahoma City, Oklahoma, United States of America; 2 Department of Microbiology and Immunology, University of Oklahoma Health Sciences Center, Oklahoma City, Oklahoma, United States of America; 3 Department of Biology, Oklahoma Christian University, Oklahoma City, Oklahoma, United States of America; University of Padova, Medical School, Italy

## Abstract

To prevent damage by reactive oxygen species, many bacteria have evolved rapid detection and response systems, including the OxyR regulon. The OxyR system detects reactive oxygen and coordinates the expression of numerous defensive antioxidants. In many bacterial species the coordinated OxyR-regulated response is crucial for *in vivo* survival. Regulation of the OxyR regulon of *Haemophilus influenzae* was examined *in vitro*, and significant variation in the regulated genes of the OxyR regulon among strains of *H. influenzae* was observed. Quantitative PCR studies demonstrated a role for the OxyR-regulated peroxiredoxin/glutaredoxin as a mediator of the OxyR response, and also indicated OxyR self-regulation through a negative feedback loop. Analysis of transcript levels in *H. influenzae* samples derived from an animal model of otitis media demonstrated that the members of the OxyR regulon were actively upregulated within the chinchilla middle ear. *H. influenzae* mutants lacking the *oxyR* gene exhibited increased sensitivity to challenge with various peroxides. The impact of mutations in *oxyR* was assessed in various animal models of *H. influenzae* disease. In paired comparisons with the corresponding wild-type strains, the *oxyR* mutants were unaffected in both the chinchilla model of otitis media and an infant model of bacteremia. However, in weanling rats the *oxyR* mutant was significantly impaired compared to the wild-type strain. In contrast, in all three animal models when infected with a mixture of equal numbers of both wild-type and mutant strains the mutant strain was significantly out competed by the wild-type strain. These findings clearly establish a crucial role for OxyR in bacterial fitness.

## Introduction

For many microbes, resistance to oxidative stress is a prerequisite for survival. This is especially so for bacteria which colonize mammals, since the host species fight infection via generation of reactive oxygen species (ROS) and reactive nitrogen species (RNS). *Haemophilus influenzae* has the human body as its sole environmental niche and thus must be able to combat the negative impact of ROS and RNS.

Commonly found as a commensal in the human nasopharynx, *H. influenzae* can also cause a diverse range of infections including otitis media, bacteremia, meningitis, epiglottitis and pneumonia [1], [2]. *H. influenzae* lacks all enzymes for the biosynthesis of porphyrin and thus has an absolute growth requirement for a porphyrin source. The porphyrin requirement can be satisfied by either heme or by protoporphyrin IX (PPIX) in the presence of iron. In order to acquire heme and iron directly from human heme-containing and/or iron-containing proteins, *H. influenzae* has evolved a complex array of mechanisms [3]–[10]. Many of these *H. influenzae* iron and heme acquisition mechanisms are tightly regulated in response to iron/heme levels [11], [12]. This tight regulation may be necessary since intracellular iron levels need to be balanced so that the redox potential of the cell is not perturbed and so that free radicals are not generated via the Fenton reaction as a consequence of excess iron in an oxidative environment [13].

*H. influenzae* possesses an inducible defense against oxidative damage mediated by OxyR [14]. The *oxyR* gene was first described in *Salmonella typhimurium and Escherichia coli* and was demonstrated to detect reactive oxygen species and coordinate the cellular response by the upregulation of almost 40 genes. These include genes encoding alkylhydroperoxide reductase, catalase, DNA binding DPS proteins, glutaredoxin, a manganese transporter and ferrochelatase [15], [16]. Orthologs of *oxyR* have since been identified among both Gram-negative and Gram-positive bacteria and play central roles in diverse metabolic activities including regulation of oxidative defenses, virulence, biofilm formation, colonization and fimbrial biosynthesis [15], [17]–[20]. The OxyR protein is a homotetramer that functions based on the redox status of two conserved cysteine residues [17]. Under oxidative stress, a disulfide bond is formed between two of the cysteine residues in each subunit of the complex, resulting in a conformational change that leads to increased affinity for binding motifs located upstream of OxyR responsive genes [21]. Binding of OxyR to its specific binding site results in transcription of the downstream gene [17], and genes controlled in this manner are designated as members of the OxyR regulon. The genes comprising the OxyR regulon vary significantly among different bacterial species; the OxyR regulons of *E. coli* and *S. enterica* comprise approximately 40 genes, in *Bacteroides fragilis* the regulon is reduced to 13 genes and in *Neisseria gonorrhea* contains only three genes [15], [22], [23]. Irrespective of the size of the regulon, regulation of a catalase gene (encoding a protein with an established role in redox homeostasis) is conserved.

The OxyR regulon has previously been defined for the nontypeable *H. influenzae* (NTHi) strain 86-028NP and was shown to comprise 11 genes [24]. Of these, the gene showing the highest fold change was that encoding the *H. influenzae* catalase, *hktE*. Also included in the NTHi 86-028NP OxyR regulon are a fused peroxiredoxin/glutaredoxin, subunits of an NAD(P) transhydrogenase, 6-phosphogluconate-dehydrogenase, a conserved ferritin-like DPS protein which has been shown to play multiple roles in stress resistance [25], as well as several proteins with a putative role in divalent metal ion acquisition (the *yfeABCD* operon). The products of the *yfeABCD* operon were originally annotated as iron uptake genes; however, it is likely that they mediate uptake of other metal ions as well. Since iron/heme homeostasis requires careful control to prevent further oxidative damage, the upregulation of proteins mediating acquisition of a potentially dangerous metal under oxidative conditions would not appear to be a sound survival strategy. It has been suggested that increases in iron and heme uptake may be due to oxidation of the intracellular iron pools leading to a transient iron starvation, or alternatively, be induced by damage to Fe-S clusters [24].

Based on previous studies of the Fe/Hm modulon [11], [12] and iron/heme stress as well as the potential for intracellular iron to impact oxidative stress, the role of OxyR in virulence and its potential role in control of iron and heme acquisition under periods of oxidative stress were investigated.

## Results

### Analysis of the OxyR Regulon Kinetics

**Figure 1:**
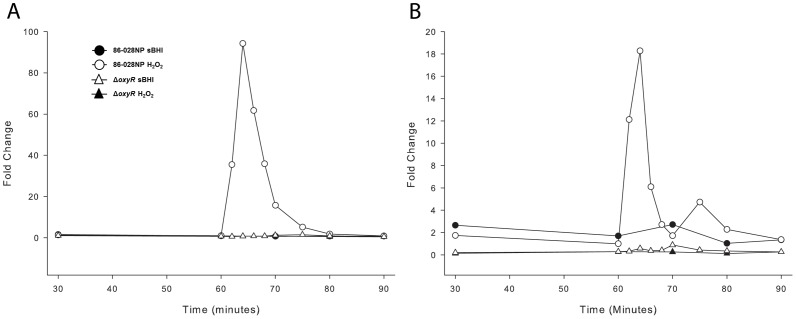
Transcriptional response of *H. influenzae* to the addition of H_2_O_2_. Comparison of transcription of the (A) *hktE* and (B) *pgdX* genes of the *H. influenzae* strain 86-028NP and the isogenic *oxyR* mutant strain HI2285. Transcriptional status of the genes was determined in strain 86-028NP(closed circles) and strain HI2285 (open triangles) grown in sBHI throughout the experiment and in strain 86-028NP(open circles) and strain HI2285 (closed triangles) grown in sBHI to which 150 µM H_2_O_2_ was added at 60 minutes.

The OxyR regulon of NTHi strain 86-028NP has previously been reported to consist of 11 genes [24]. Initially experiments were performed to determine the kinetics of regulation of members of the OxyR regulon in response to oxidative stress. For each *H. influenzae* isolate, two flasks were inoculated to give ∼2×10^8^ CFU/ml. After 60 minutes of culture, one of the two flasks was supplemented with 150 µM H_2_O_2_ and incubation continued for a further 20 minutes. Samples were taken from both flasks to determine RNA profiles and viable counts at specified times over the course of the experiment. Since catalase (*hktE*) was reported as the gene with the highest fold change under oxidative stress in *H. influenzae* [24], this gene was selected as a marker for assessing the kinetics of the regulon. Upon addition of the perturbant to a growing culture of the NTHi isolate 86-028NP, there was an immediate increase in the abundance of *hktE* transcripts such that within four minutes the transcript level was approximately 100-fold greater than that of the pre-treatment sample (Fig. 1). The level of *hktE* transcripts peaked between 4 and 6 minutes after H_2_O_2_ addition and decreased to basal levels over a further 15 minutes (Fig. 1). In the control flask (no H_2_O_2_ addition), *hktE* transcripts remained constant (Fig. 1). Similar kinetics were observed with both the *H. influenzae* type b (Hib) strain 10810 and strain Rd KW20, although peak fold changes for both strains were lower than those observed with 86-028NP (52 and 50-fold for strains 10810 and Rd KW20 respectively). Each strain remained viable over the time course of the experiment (data not shown). To confirm that this kinetic profile was not restricted to *hktE*, profiles were also examined for the peroxiredoxin/glutaredoxin gene *pgdX*. Kinetic profiles for *pgdX* were similar to those reported for *hktE*, although the magnitude of induction was lower. Strain 86-028NP exhibited a maximum peak value of 18-fold, while values of 30 and 9-fold were observed for strains Rd KW20 and 10810 respectively.

### Impact of Mutation of the *oxyR* Gene on in vitro Growth

Having established a reliable assay to determine the kinetics of regulation the *oxyR* gene was deleted from the *H. influenzae* genome in order to confirm that catalase would become unresponsive to oxidative stress. Deletions of *oxyR* were constructed in each of the three *H. influenzae* strains 10810, 86-028NP and Rd KW20 and the mutants were designated HI2283, HI2285 and HI2274 respectively.

**Figure 2:**
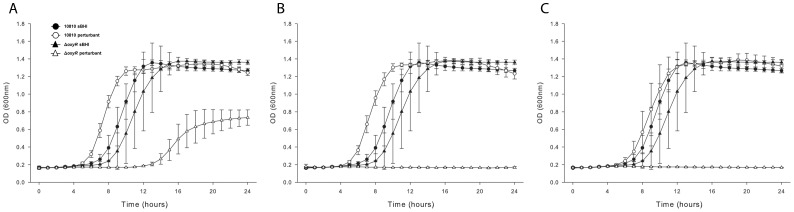
Growth of *H. influenzae* strains in sBHI supplemented with one of three oxidizing agents. Growth of *H. influenzae* type b strain 10810 and the isogenic *oxyR* mutant HI2283 when supplemented with (A) 150 µM H_2_O_2_, (B) 100 µM cumene hydroperoxide and (C) 100 µM Tert-butyl peroxide. In each panel growth in sBHI is represented by closed circles for 10810 and by closed triangles for HI2283, while growth in sBHI supplemented with the specified perturbant is represented by open circles for strain 10810, and by open triangles for strain HI2283. Values are mean±SD for quintuplicate results from representative experiments. The Mann-Whitney test was used to compare growth of strain10810 and HI2283 over the entire growth period for each perturbant. For each perturbant, the growth of the mutant strain was statistically different from growth of the wild-type strain (*P*<0.0001 for all analyses).

Initially the respective *oxyR* mutants of each isolate were compared to the corresponding wild type isolate for any impact on resistance to oxidative damage. Figure 2 shows representative growth curves for strain 10810 and its isogenic *oxyR* mutant, HI2283. In the absence of oxidative stress, there was no significant difference between the *oxyR* mutant HI2283 and the wild-type strain. However, compared to the wild-type strain the mutant exhibited increased sensitivity to each oxidizing agent examined (Fig. 2). Similar results were seen in both strains NTHi 86-028NP and Rd KW20 (data not shown). Complementation of the *oxyR* mutation restored resistance to peroxide in the *oxyR* mutant strains (data not shown).

### Catalase and Peroxiredoxin Regulation in an *oxyR* Mutant Background

Once mutants of *oxyR* were confirmedthe effect of the mutation on transcriptional regulation of members of the reported OxyR regulon was investigated. In the absence of perturbant, the basal level transcripts of genes *hktE* and *pgdX* in the *oxyR* mutant were depressed below the level of transcripts in the wild-type progenitor strain 86-028NP (Fig. 1). While transcript levels for both genes are shown, the lower basal levels of transcripts in the *oxyR* mutant are more clearly apparent for *pgdX* transcripts (Fig. 1B) than for the *hktE* transcripts (Fig. 1A) due to the scaling of the respective graphs. The mutant had approximately 5 to 10-fold less basal levels than the wild-type strain for both *hktE* and *pgdX,* and neither gene responded to the addition of H_2_O_2_ in the mutant strain (Fig. 1). Similar results were observed when strains 10810 and Rd KW20 were examined (Table 1). These data demonstrate that the increase in *hktE* and *pgdX* transcripts observed in the wild-type strains in response to H_2_O_2_ is due to the action of OxyR.

**Table 1:** Comparison of the fold change of the members of the OxyR regulon across several isolates of *H. influenzae* following peroxide treatment.

Gene^a^	NTHi 86-028NP			Hi Rd KW20		Hib 10810	
	MA^b^	WT^c^	ΔOxyR^d^	WT	ΔOxyR	WT	ΔOxyR
*hktE*	61	140.5	1.02	52.81	1.29	59.2	0.56
*pgdX*	7	19.4	0.64	5.79	0.28	5.4	0.1
*Gnd*	4	3.7	0.73	1.42	0.8	2.8	0.38
*Dps*	4	24.6	0.96	46.81	0.47	20.17	0.13
*yfeB*	7	8.5	1.53	2.42	1.11	1.21	0.48
*pntA*	2	4.7	0.29	2.33	0.47	4.32	0.32

a Gene being studied. In instances where an operon is part of the OxyR regulon, a single gene was examined, as indicated.

b Results from the previously published microarray (MA) work of Harrison *et al*.

c Fold change of the pre and post addition of peroxide in flasks containing the wild-type isolate.

d Fold change of the pre and post addition of peroxide in flasks containing the OxyR mutant.

### Comparison of the OxyR regulon between *H. influenzae* Strains

Previously, the OxyR regulon has been reported for the nasopharyngeal NTHi isolate 86-028NP and shown to contain seven distinct operons [24]. The OxyR regulon was examined in several *H. influenzae* isolates in order to identify the genes defining the core regulon. In addition, to confirm that the genes were under control of OxyR, the *oxyR* mutant of each isolate was also examined. All of the loci previously identified in strain 86-028NP as OxyR-regulated were found to be responsive to the addition of H_2_O_2_ in the current study (Table 1). However, differences in the transcriptional profiles of these genes were observed among the strains. In Rd KW20, the *gnd* gene was not regulated by exposure to H_2_O_2_, while in the isolate Hib 10810 the *yfeABCD* operon was non-responsive. The gene NTHI0684 (HI0555) identified in the previous study as regulated was not examined in this study as it is likely a pseudogene. In 86-028NP the NTHI0684 CDS encodes approximately 60 amino acid residues, while in 10810, Rd KW20 and other isolates there is an N terminal extension of 20 or more residues and in the NTHi isolate 6P18HI there are both C and N terminal extensions in excess of 110 residues. The putative CDS also contains various frame shifts in different isolates and, in numerous other isolates (R2846, R2866 and others), the gene is missing from the genome. Harrison *et al.* also reported that a number of other operons were apparently indirectly regulated by oxidative stress including several genes involved with iron and heme homeostasis [24]. In addition to the 7 operons directly responsive to OxyR, the transcriptional response to H_2_O_2_ of several of these genes, previously identified as responsive to oxidative stress in an OxyR-independent manner, was also examined. Specifically, transcripts of the genes *exbD, hitA* and *tbp1* were examined. None of these genes showed a significant level of upregulation following exposure to peroxide. As a control, several genes with no association to *oxyR* were examined. None showed significant change following exposure to oxidants in either wild-type or mutant strains (data not shown).

### Catalase and Peroxiredoxin Regulation Kinetics

**Figure 3:**
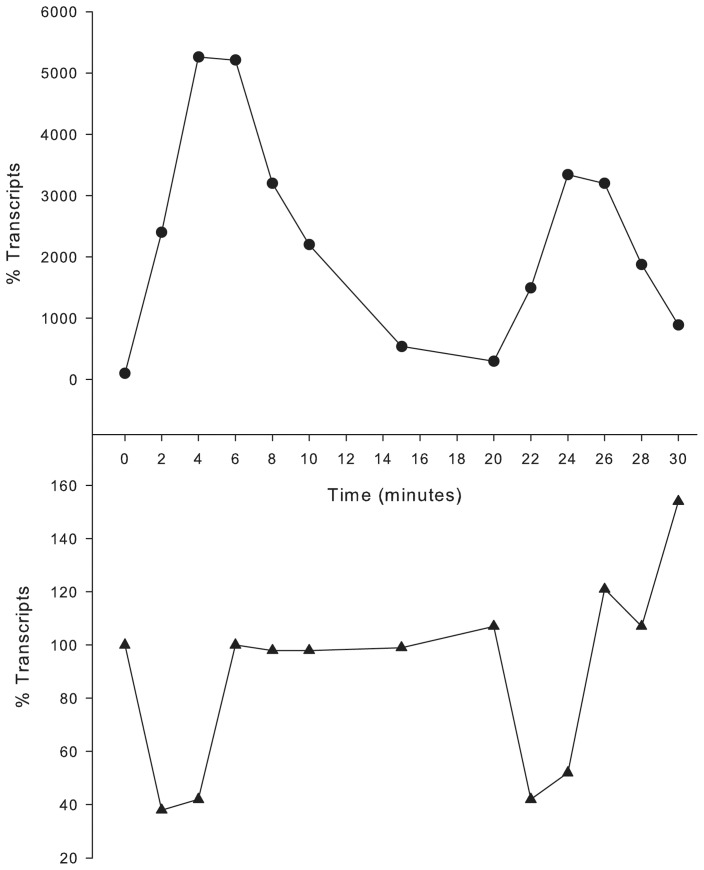
Transcriptional response of *H. influenzae* to sequential additions of H_2_O_2_. Percent change in transcript levels of the *hktE* (closed circles) and *oxyR* (closed triangles) upon addition of H_2_O_2_. *H Influenzae* strain 86-028NP was grown in sBHI for 60 minutes at which point 150 µM H_2_O_2_ was added (corresponds to time 0 in the graph above), after a further 20 minutes of growth a second addition of 150 µM H_2_O_2_ was made.

An interesting aspect of the kinetics of gene transcription in response to peroxide addition was the very rapid decrease in transcripts a few minutes after exposure to the perturbant (Fig. 1). This phenomenon may be due to either the relaxation of oxidative stress or, alternatively, a negative regulatory event. Due to the rapidity of the observed decrease, it is possible that a negative regulator acts to suppress the continued elevated transcription of *hktE* and *pgdX*. To address this possibility, experiments were performed in which cultures were challenged twice with H_2_O_2_, the second exposure occurring after the apparent decrease in transcription. Both additions of H_2_O_2_ were equivalent and spaced at 20 minute intervals. Transcript levels of both *hktE* and *pgdX* following the primary peroxide challenge closely matched those seen in previous experiments (Fig. 3 shows data for *hktE* transcripts only; *pgdX* transcripts showed a similar pattern). After the second challenge, transcripts again increased rapidly exhibiting a similar kinetic profile to that seen following the primary challenge (Fig. 3). However, for both *hktE* and *pgdX*, the magnitude of the increase in transcripts following the second challenge was significantly lower than that observed following the initial challenge (Fig. 3). This latter observation indicates that the second response is being potentiated, or partially inhibited. Examination of the half-life of the transcripts showed that the rate of decay was the same for the primary and secondary challenges (data not shown). In addition to the transcriptional changes of *pgdX* and *hktE*, the transcriptional changes in *oxyR* were also examined. As *pgdX* or *hktE* transcripts rise the transcripts of *oxyR* decrease and conversely as *pgdX* and *hktE* transcripts decrease the *oxyR* transcripts rise (Fig. 3). This may indicate the steric hindrance of *oxyR* transcription by OxyR binding to the upstream of *pgdX*, since *pgdX* and *oxyR* are both transcribed from a divergent promoter region. It also provides evidence that the drop in transcripts of *pgdX* and *hktE* is preceded by release of the bound OxyR. This simple model does not however account for the apparent potentiation of signal seen during the second challenge. Since the abrogation of signal is apparent in the second challenge, it is reasonable to suggest that the factor responsible for this reduction is induced as part of the initial OxyR response to the first challenge.

### Negative Control of Expression via OxyR-induced PgdX

**Figure 4:**
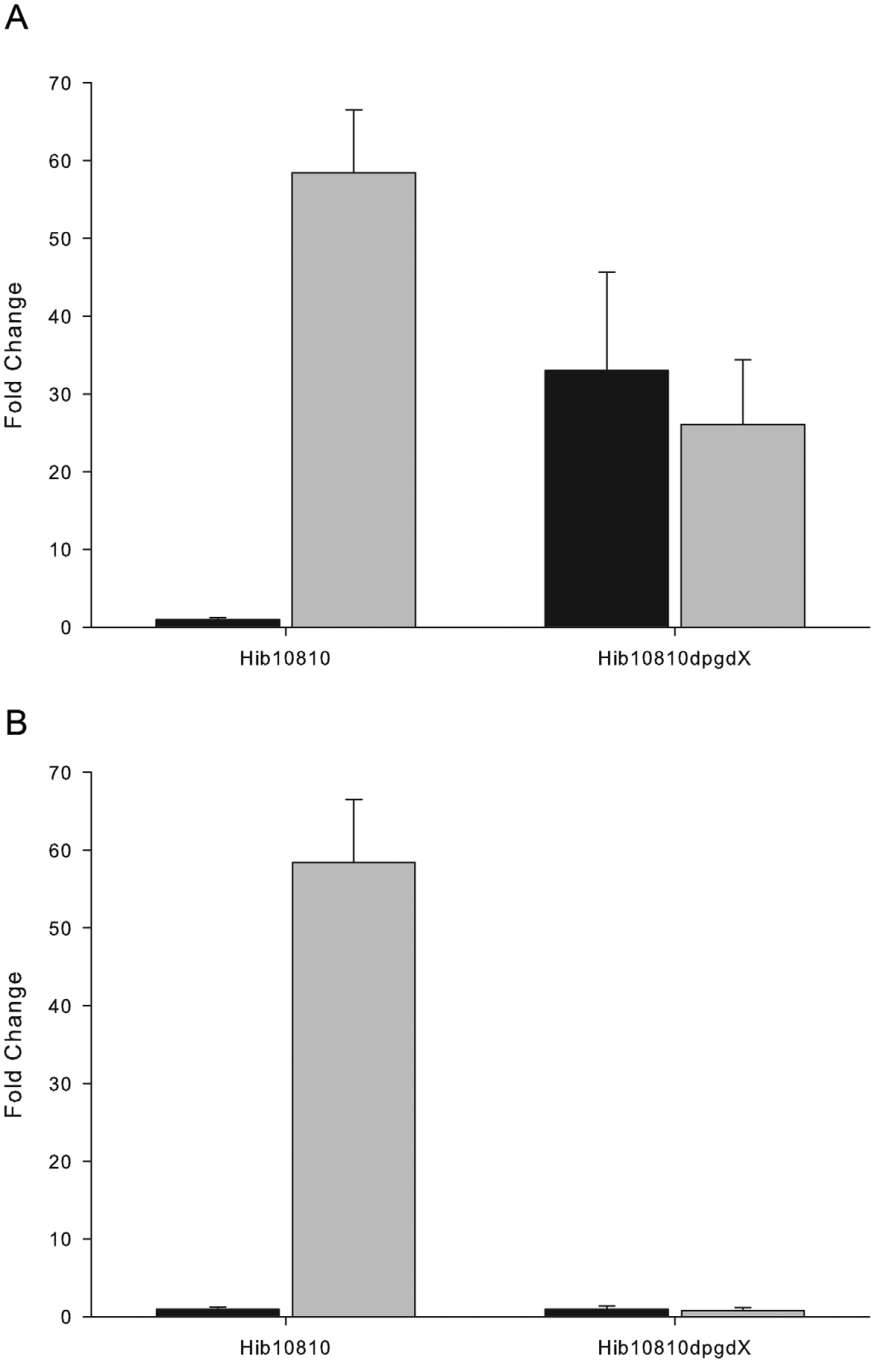
Transcriptional response of an *H. influenzae* pgdX mutant to the addition of H_2_O_2_. Comparison of transcription of the *hktE* gene of the *H. influenzae* strain 10810 and the isogenic *pgdX* mutant strain HI2159. *H. influenzae* was grown for 60 minutes in sBHI and then supplemented with 150 µM H_2_O_2_. Transcript levels of *hktE* were determined on two samples, one taken immediately prior to H_2_O_2_ addition (black bars) and the second taken 4 minutes after H_2_O_2_ addition (grey bars). (A) *hktE* transcript levels of individual strains are normalized to the pre-addition transcript level of that specific strain. (B) all *hktE* transcript levels are normalized to the pre-addition transcript level of strain 10810.

During oxidative stress, conserved cysteine residues on OxyR become oxidized which leads to activation of the protein complex; oxidized OxyR initiates upregulation of the members of the OxyR regulon [17]. The data reported herein demonstrate a rapid onset of upregulation of members of the OxyR regulon, followed by a rapid decrease in transcripts. In addition, transcript levels following a secondary challenge with an oxidant reveal potentiation of the upregulation. This is presumably due to the reduction of the conserved cysteine residues in OxyR, leading to an inactive OxyR protein complex with concomitant release of the bound DNA. The fused peroxiredoxin/glutaredoxin (*pgdX*) located adjacent to *oxyR* in the *H. influenzae* genome and a member of the OxyR regulon, may play a role in this process. To address this possibility a mutant lacking *pgd*X was created and the transcriptional response of the *hktE* gene to challenge with H_2_O_2_ was examined. Transcript levels of *hktE* were insensitive to oxidant challenge in the *pgdX* mutant background (Fig. 4). However, *hktE* transcripts in the *pgdX* mutant were elevated by approximately 30-fold over *hktE* transcripts in the unstressed wild-type strain. While this 30-fold elevation is lower than the elevation shown in Figure 1, this value is consistent with other fold changes of induction of this gene and may be lower due to minor variations in the time of sampling, which, based on the steep slope seen in the response profile, could have a dramatic effect on experimentally determined transcript levels. These data demonstrate that even in the absence of oxidant, the catalase gene is being transcribed in the *pgd*X mutant. Presumably this is achieved through the action of an oxidized OxyR complex, suggesting that a functional PgdX is required for the reduction of activated OxyR. Without the reduction of OxyR by PgdX, the OxyR complex remains oxidized and, thus, insensitive to the assault by peroxide. It is known that aerobic metabolism can generate sufficient oxidants to activate OxyR [16].

### Impact of the *oxyR* Mutation in Animal Models of Disease

To assess the potential contribution to virulence of the *oxyR* gene, *oxyR* mutant strains were compared to their corresponding wild-type strains in multiple animal models of *H. influenzae* disease. Initially the *oxyR* mutant of the Hib strain 10810 was compared to its wild-type progenitor in 4 different experiments using rat models of bacteremia.

**Figure 5:**
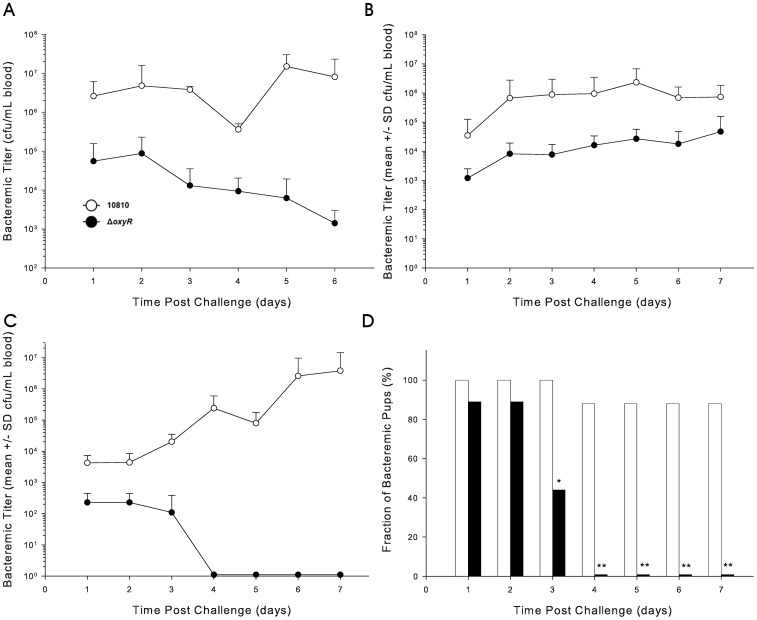
Impact of the *oxyR* mutation in rat models of virulence and competitive fitness. Bacteremic titers in (A) 5-day old rats and (B) 30-day old rats infected with a mixture of equal numbers of the wild-type Hib strain 10810 (open circles) and the *oxyR* mutant strain HI2283 (closed circles). (C) Bacteremic titers in 30-day old rats infected with a mixture of equal numbers of the wild-type Hib strain 10810 (open circles) and the *oxyR* mutant strain HI2283 (closed circles). Results for bacteremic titers are all means±SD from groups of 10 rats. (D) Percentage of freshly drawn blood samples from 30-day old rats infected with a mixture of equal numbers of the wild-type Hib strain 10810 and the *oxyR* mutant strain HI22283 containing detectable colonies of the wild-type strain (black columns) or the mutant strain (white columns). (^*^ *P* = 0.021, ^**^ *P*<0.002,).

In the first experiment two groups of 5-day old infant rats were intraperitoneally injected with either strain 10810 or the isogenic *oxyR* mutant HI2283 in order to compare virulence of the two strains. There was no significant difference in bacteremic titers or in clearance between the two groups over the 6-day course of the experiment (data not shown). In the second experiment a group of rats was infected with equal numbers of both the wild-type strain 10810 and the oxyR mutant to assess the competitive fitness,and titers of the mutant strain were significantly lower than those of the wild-type strain on all days (Fig. 5).

The virulence and fitness comparisons of strain 10810 and its *oxyR* mutant were repeated in 30-day old weanling rats. Previously, significant differences in the impact of numerous mutations on virulence when comparing 5-day old to 30 day-old rats have been reported [6], [7], [26]–[28]. When two groups of 30-day old rats were infected with either the wild-type or the mutant strain, the group infected with the *oxyR* mutant had consistently lower bacterial titers than the group infected with the wild-type strain (Fig. 5). In the fitness study, where equal numbers of wild-type 10810 and its *oxyR* mutant are co-inoculated, titers of the mutant strain were significantly lower than those of the wild-type strain on days 1–3 post infection (Fig. 5). On day 3 post infection the wild-type strain was detectable in all rats while the mutant strain was detectable in only 40% of rats (*P* = 0.02), and on days 4–7 post infection the mutant had cleared from all animals while the wild-type remained detectable in 90% (*P* = 0.0007) (Fig. 5).

Additionally the *oxyR* mutant of the NTHi strain 86-028NP was compared to its wild-type progenitor in chinchilla models of otitis media in two separate experiments. In the first experiment two groups of 5 chinchillas were transbullarly infected with either strain 86-028NP or the isogenic *oxyR* mutant HI2285 (actual infective doses were 753 CFU for 86-028NP and 879 CFU for HI2285). There were no significant differences between bacterial titers in middle ear effusions (MEE) over the 22 days of the experiment (data not shown).

**Figure 6:**
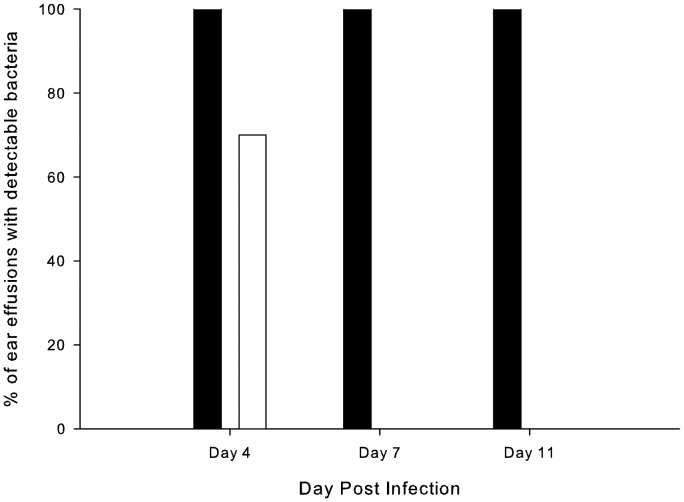
Impact of the *oxyR* mutation in a chinchilla model of competitive fitness. Percentage of successfully tapped ears infected with a mixture of equal numbers of the wild-type strain 86-028NP and the *oxyR* mutant strain HI2285 containing detectable colonies of the wild-type strain (black columns) or the mutant strain (white columns). Using Fisher’s Exact test to compare percentages of effusions with detectable wild-type or mutant strains *P*<0.0001 on both day 7 and day 11.

In the second experiment a group of 5 chinchillas was infected with a mixture of equal numbers of both the wild-type strain 86-028NP and the isogenic *oxyR* mutant (the actual infective dose was 2130 CFU equally divided between the two strains). The *oxyR* mutant strain was rapidly cleared from the ear while the wild-type persisted. On day 4 post infection the wild-type strain was detectable in MEE from all ears while the mutant strain was absent in 3 of the 10 MEE (Fig. 6). On day 4 titers for the wild-type strain averaged 1.1×10^8^ CFU/ml (±1.4×10^8^ CFU/ml) while those for the *oxyR* mutant averaged 3.3×10^4^ CFU/ml (±1×10^5^ CFU/ml) (*P*<0.0001). On days 7 and 11 all ears were cleared of the mutant strain while the wild-type remained present in all ears (Fig. 6), for example on day 11 titers for the wild-type strain averaged 6.8×10^6^ CFU/ml (±1.3×10^7^ CFU/ml).

### Impact of the *pgdX* Mutation in Animal Models of Disease

**Figure 7:**
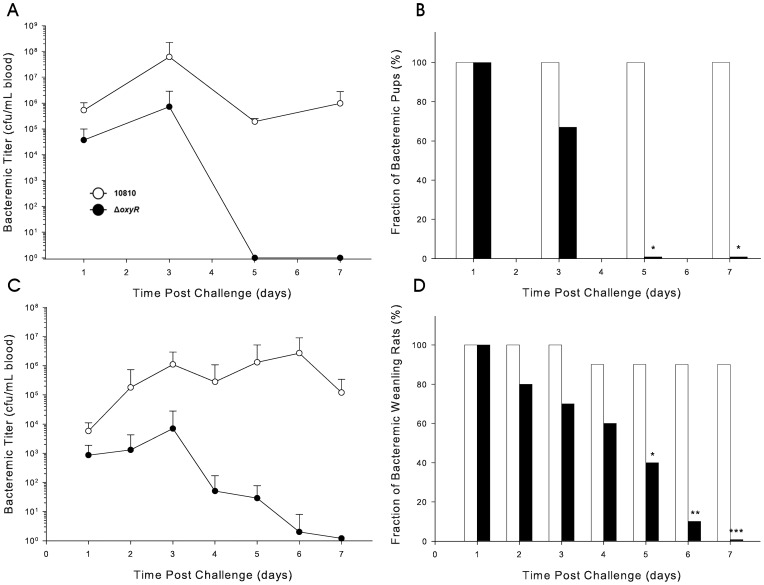
Impact of the *pgdX* mutation in rat models of competitive fitness. Bacteremic titers in (A) 5-day old rats and (C) 30-day old rats infected with a mixture of equal numbers of the wild-type Hib strain 10810 (open circles) and the *pgdX* mutant strain HI2159 (closed circles). Results are means±SD from groups of 10 rats. Percentage of freshly drawn blood samples from (B) 5-day old rats and (D) 30-day old rats infected with a mixture of equal numbers of the wild-type Hib strain 10810 and the *pgdX* mutant strain HI2159 containing detectable colonies of the wild-type strain (black columns) or the mutant strain (white columns). (^*^ *P*<0.005, ^**^ *P* = 0.005, ^***^ *P*<0.0001).

The contribution of the *pgdX* gene to both virulence and fitness was also examined in the infant and weanling rat models of bacteremia in a series of four experiments identical to those used to assess the *oxyR* mutant. I Neither of the virulence studies in either 5-day old or 30-day old revealed any significant differences in bacterermic titers or clearance between the cohorts infected with wild-type and mutant strains (data not shown). In contrast, there was a significant difference in the bacteremic titers of the two strains following co-infection of either 5-day old or 30-day old rats with equal numbers of both the wild-type strain and the *pgdX* mutant (Fig. 7).

**Figure 8:**
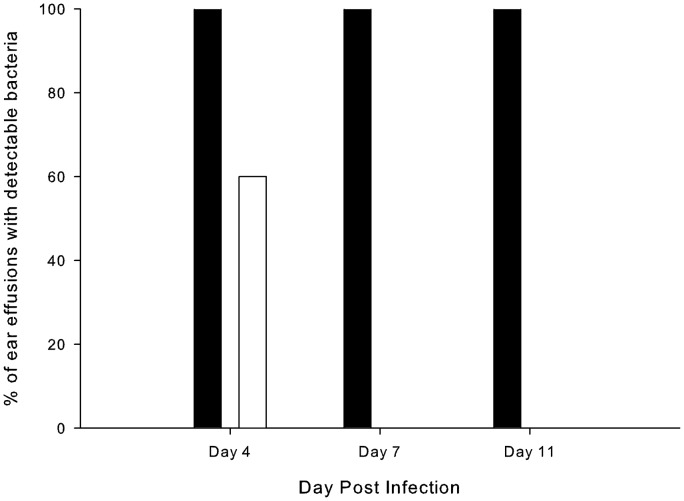
Impact of the *pgdX* mutation in a chinchilla model of competitive fitness. Percentage of successfully tapped ears infected with a mixture of equal numbers of the wild-type strain 86-028NP and the *pgdX* mutant strain HI2334 containing detectable colonies of the wild-type strain (black columns) or the mutant strain (white columns). Using Fisher’s Exact test to compare percentages of effusions with detectable wild-type or mutant strains *P* = 0.0002 on day 7 and *P* = 0.022 on day 11.

The pgdX mutant of 86-028NP was also assessed in the chinchilla model of otitis media. When 2 groups of chinchillas were infected with either the wild-type NTHi strain 86-028NP or its isogenic *pgdX* mutant HI2334 (actual infective doses were 500 CFU for 86-028NP and 200 CFU for HI2334) there were no significant differences bacterial titers within recovered MEE or clearance over the 18 days of the experiment (data not shown). Conversely, when 5 chinchillas were infected with equal numbers of the wild-type strain and the *pgdX* mutant (actual infective dose 1230 CFU equally divided between the two strains), the wild-type strain persisted through the course of the experiment while the mutant strain was cleared by day 7 (Fig. 8). The average titers of the two strains in MEE recovered at day 4 were significantly different with the wild-type being present at a titer of 9.2×10^7^ CFU/ml (±1.2×10^8^ CFU/ml) and the mutant strain at 106 CFU/ml (±162 CFU/ml) (P<0.0001).

### Transcription of the OxyR Regulon Genes during Experimental Otitis Media

**Figure 9:**
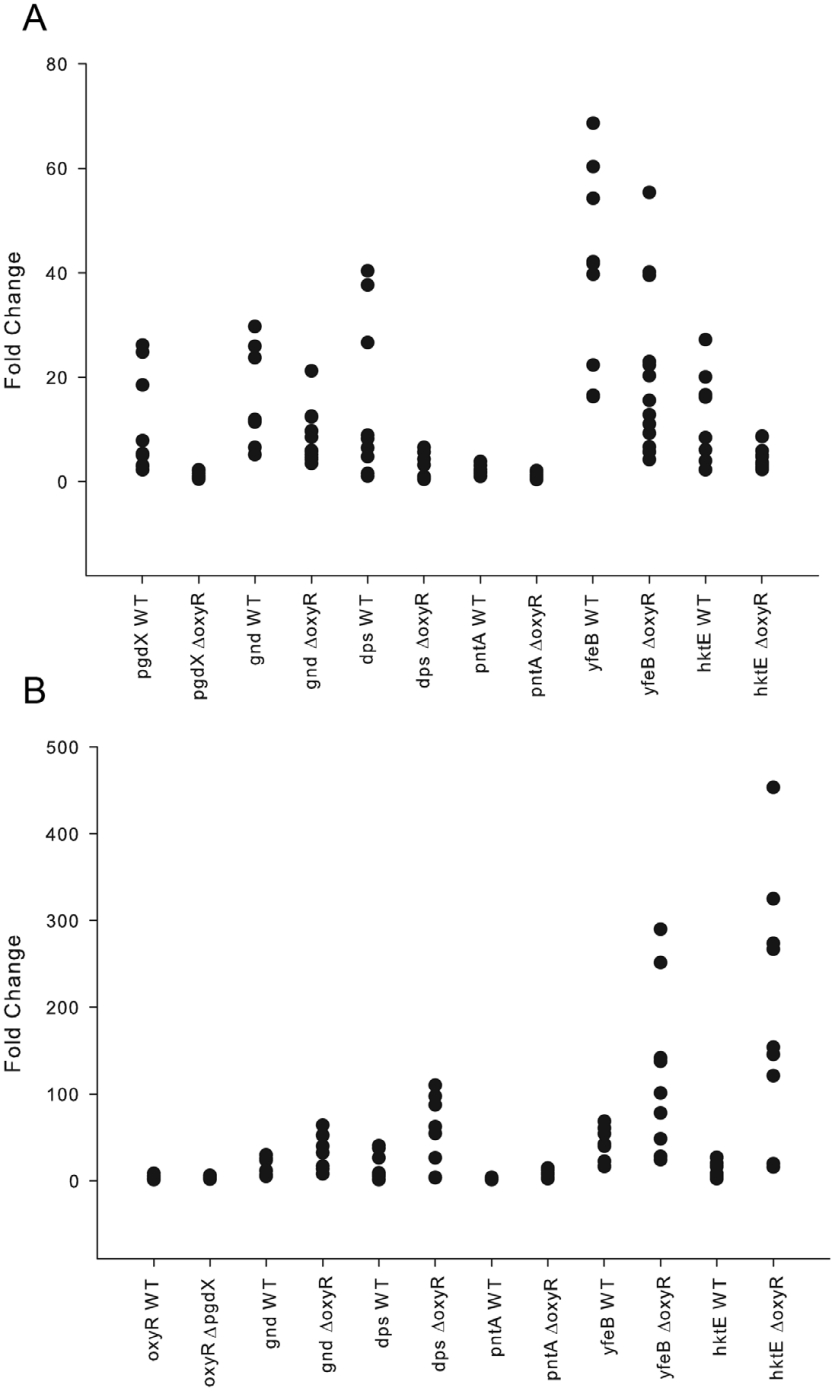
Transcription of genes in the *H. influenzae* OxyR regulon *in vivo*. Comparison of transcript levels of the individual members of the OxyR regulon during colonization of the chinchilla ear with (A) either the wild-type NTHi strain 86-028NP or the *oxyR* mutant strain HI2285 (ΔoxyR) and (B) with either the wild-type NTHi strain 86-028NP or the *pgdX* mutant strain HI2334 (ΔpgdX). Data points represent Q-PCR values of fold change in transcripts by comparison with non-stressed *in vitro* grown 86-028NP.

The transcriptional status of the genes comprising the OxyR regulon was also determined during experimental infection of the chinchilla ear with NTHi 86-028NP. To determine the degree of expression, the samples were normalized to the fully repressed *in vitro* sample of 86-028NP. Among members of the OxyR regulon, *hktE, gnd, dps, yfeB* and *pgdX* were upregulated on all days of infection (Fig. 9A). In contrast, transcript levels of *pntA* were reflective of the uninduced levels seen *in vitro*. *In vivo* transcript levels of the genes in the OxyR regulon were also determined in MEE samples from chinchillas infected with either the *oxyR* mutant strain or the *pgdX* mutant strain. In samples from animals infected with the *oxyR* mutant only *gnd* and *yfeB* were expressed above the basal level observed *in vitro* (Fig. 9A). In samples from animals infected with the *pgdX* mutant *hktE*, *yfeB* and *dps* were significantly upregulated compared to transcript levels in samples from wild-type infected animals (Fig. 9B).

## Discussion

Growing within the human host, *H. influenzae* is assaulted by oxidative agents, and must detect and detoxify these potentially lethal oxidants if it is to survive and proliferate. *H. Influenzae* upregulates expression of several gene products in response to ROS exposure, and these are regulated solely by OxyR. The OxyR regulon of *H. influenzae* contains catalase, a fused peroxiredoxin/glutaredoxin (PgdX), a DNA binding (ferritin like) protein (Dps), an NAD(P) transhydrogenase (PntAB), a putative metal ion transport system encoded by the *yfeABCD* operon, and a 6-phosphogluconate-dehydrogenase (Gnd) [24]. With the exception of YfeABCD all of the above gene products have a demonstrated role in oxidative resistance [25], [29]–[31].

The contributions to virulence of some of the individual members of the OxyR regulon have been investigated in different isolates of *H. influenzae* [29], [31], [32]. Studies on catalase deficient mutants in the isolates Eagan and Rd KW20 demonstrated increased sensitivity to peroxides of the isogenic isolate by comparison with the wild-type, however, in comparative virulence studies the *hktE* mutant of the strain Eagan showed no significant difference in virulence compared to the wild-type [29]. In separate studies both a *pgdX/hktE* double mutant of strain Eagan and a *dps* mutant of strain Rd KW20 were unaffected in virulence by comparison with the wild-type strains [31], [32]. In the case of the *pgdX*/*hktE* double mutant this failure to affect virulence was despite all peroxiditic activity being abrogated [31]. The current study, sought to expand upon these observations by examining the OxyR regulon in several isolates, whether the genes are expressed *in vivo* and if OxyR is required for virulence in these isolates.

In initial experiments to determine the kinetics of genes in the OxyR regulon an almost immediate increase in transcripts of the catalase gene, *hktE*, by over 50-fold within 4 minutes of addition of H_2_O_2_ was observed, followed by a rapid decline with the transcript levels dropping to the uninduced level within approximately 20 minutes. Additional experiments with sampling at 15 second intervals showed that the response was initiated within 30 seconds with a peak increase in *hktE* transcripts of approximately 400-fold (data not shown). In contrast, an *oxyR* mutant did not show an increase in transcripts in response to peroxide addition, but exhibited a steady state level reduced from that of the wild-type. This latter observation indicates a constitutively low level of OxyR mediated induction of these genes, presumably due to oxidation of reduced OxyR (OxyR^r^) to the DNA- binding oxidized OxyR (OxyR°). Low level activation has been previously posited as part of the resistance to ROS generated intrinsically by oxidative respiration [33].

Comparison of the strain 86-028NP OxyR regulon with the regulons of two additional strains demonstrated that *hktE*, *pgdX*, the DPS protein gene and the genes encoding the NAD(P) transhydrogenase were all upregulated following oxidative stress (Table 1). The 6-phosphogluconate dehydrogenase gene was upregulated in 86-028NP and in the Hib strain 10810 but was not regulated in strain Rd KW20. Conversely *yfeABCD* was upregulated in both strains 86-028NP and Rd KW20, but was insensitive to oxidative stress in strain10810 (Table 1). These data reveal isolate specific variation in the response to oxidative stress and indicate that additional genes, not identified in the original 86-028NP analysis, may be involved in the global response of oxidative stress in *H. influenzae*. Identification of additional oxidant-responsive genes would require global transcriptomic analyses to be performed with several additional *H. influenzae* isolates.

The lack of regulation of the *yfeABCD* operon in the Hib strain 10810 is of particular interest since we have previously shown this operon to be insensitive to iron/heme stress in this isolate. In contrast, transcription of *yfeABCD* was induced in several other *H. influenzae* strains under identical conditions of iron/heme restriction [11], [12]. The raw data for *yfeABCD* transcripts produced herein for strain 10810 indicate that even though the gene is not regulated, it is constitutively expressed at a level consistent with “up-regulation” observed in the other isolates (data not shown). Thus, in this case lack of regulation of the *yfeABCD* operon in 10810 does not indicate lack of expression. Comparison of the upstream region of the *yfeABCD* among isolates shows a single base pair difference in a region with homology to a putative Fur (ferric uptake regulator) binding site located approximately 100 bases from the translational start. This change in the sequence of the proposed Fur-binding site may explain the apparent regulatory differences. The distance of this potential Fur-binding site from the start of the operon indicates a possible role for the long untranslated region as a regulatory element. Preliminary analyses using secondary structure predictions of this region indicate a high degree of structure consistent with this hypothesis (data not shown). Previous studies demonstrating iron/heme regulation of the *yfeABCD* operon indicate that it may have a role in iron homeostasis [11], [12]. However, based on OxyR regulons of other bacteria, *yfeABCD* may be associated with manganese uptake. In the OxyR regulons of *E. coli*, *S. typhimurium* and *Shigella flexneri*, MntH is responsible for the import of manganese to modulate the intracellular metal environment and prevent deleterious Fenton reactions from occurring [16], [34], [35]. Based on homology, regulation by OxyR, and the need to limit iron uptake, the *yfeABCD* operon may play an analogous role in *H. influenzae*.

**Figure 10:**
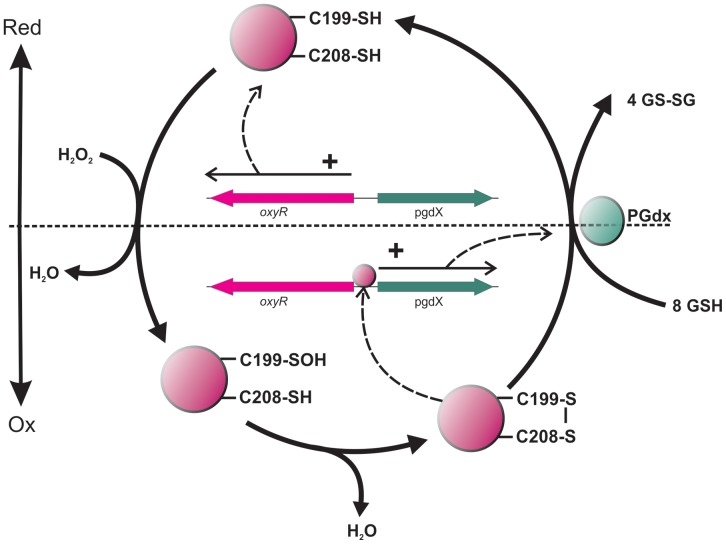
Schematic of the proposed regulation of the *H. influenzae* OxyR system. The *oxyR* (pink arrow) and *pgdX* (green arrow) genes are divergently transcribed from the same intergenic region. Under reducing conditions, OxyR is synthesized *de novo* with reduced disulfides at positions 199 and 208. On exposure to elevated peroxide levels, these reduced disulfides are sequentially oxidized to create a disulfide bridge. The oxidized OxyR is able to bind to promoters upstream of members of the OxyR regulon. As a result of OxyR binding to the promoter the transcription of *pgdX* increases. PgdX acts to reduce the disulfides of OxyR, releasing it from the promoters and halting further transcription. In addition, binding of OxyR to the *pgdX* promoter temporarily sterically inhibits transcription and further *de novo* synthesis of OxyR. The dotted line represents the redox status of the cell, with the upper section of the figure representing reducing conditions and the lower section representing oxidizing conditions.

The kinetics of transcript levels for members of the OxyR regulon following exposure to H_2_O_2_ showed a rapid decrease in transcripts beginning at approximately 6 minutes post exposure. This led us to speculate a negative aspect of regulation by OxyR that was controlling the long term transcription of these genes. To address this, an experiment was performed with two additions of peroxide, the second timed at 20 minutes after the initial dose (when transcripts had returned to the basal level). There was a markedly reduced fold increase in transcripts of both *hktE* and *pgdX* in response to the second dose, although the overall profile remained the same (Fig. 3). The similar rate of transcript decay seen following both the primary and secondary H_2_O_2_ additions is presumably due to a similar half-life of the transcripts in both cases. This indicates that the potentiation of the fold change is a direct consequence of a lower level of induction, rather than a more rapid degradation of the message. Thus, the potentiating agent may be induced by oxidative stress and may itself be a member of the OxyR regulon. Since OxyR exerts its effect by conformational change following reversible disulfide bridge formation [17], the *pgdX* gene product would be an ideal candidate for this potentiating agent. The *pgdX* encoded protein is a fused peroxiredoxin/glutaredoxin with the ability to reduce the disulfide bridges at the expense of glutathione. Mutation of *pgdX* abrogated the *hktE* response to oxidative stress in all of the tested *H*. *influenzae* isolates. Transcript levels of *hktE* in the *pgdX* mutants, both prior to and following H_2_O_2_ addition, were consistent with levels observed in fully induced wild-type cells, indicating that OxyR is oxidized and actively binding the *hktE* promoter. The oxidized status of OxyR in these circumstances is presumably due to the low level of endogenous peroxide produced by general metabolism. It has been previously shown that the metabolism of *H. influenzae* and other organisms may generate peroxide at approximately 1-12 µm/sec [30], [36]. There are two possible explanations for the apparent constitutive expression of the OxyR regulon in the *pgdX* mutant; 1) PgdX directly reduces the level of oxidant and thus decreases the oxidative stress below a threshold level resulting in oxidized and activated OxyR or 2) PgdX directly reduces OxyR° and thus turns off oxidative induced gene upregulation. The latter model is favored, since the oxidized thiols of OxyR° are substrates of PgdX homologs in other species [17], [33] and, being regulated directly by OxyR, the increase in PgdX would be delayed from the initial oxidative attack and control the long term expression of catalase and the other members of the OxyR regulon. By such coordinated regulation, OxyR may effectively act in a dose dependent manner by balancing the oxidation (by the perturbant) and subsequent reduction (*via* the induced PgdX) of the thiols on its surface. In this manner the response to oxidative stress can be tightly controlled at a level proportionate to the stress. Previous studies on the *H. influenzae pgdX* gene demonstrated that *pgdX* mutants in strain Rd KW20 also showed an increased level of catalase activity [30], [37]. The authors concluded that *pgdX* was required for scavenging low levels of endogenous peroxides, and proposed that in the *pgdX* mutants these endogenous peroxides increased beyond the threshold level required to activate production of catalase. However, based on our data, which indicates that the OxyR mediated response to oxidative stress is finite and short lived, once the increased catalase had reduced all the peroxide the oxidative stress should be removed, and OxyR° would return to a reduced form and the induction of catalase would cease. Thus the increased expression of catalase in a *pgdX* mutant would not be accounted for by the previously proposed mechanism. However, the requirement for reduction of OxyR° by PgdX would account for the observed stable increase in catalase activity observed in *pgdX* mutants. These studies also show a decrease in *de novo* transcription of *oxyR* following oxidative stress, possibly by OxyR° causing steric hindrance of the *oxyR* promoter by binding at the *pgdX* promoter. While *oxyR* transcripts show a 50% reduction they rapidly return to the basal level within 4 minutes post stress; this return to the basal levels corresponds to the release of OxyR from the *pgdX* promoter. The schematic, shown in figure 10 and based on the previous model of Lushchak [38], summarizes this and proposes a cyclical pathway of OxyR regulation. Such a model would allow for fine tuning of redox homeostasis and indicates a constant dynamic flux of reduced and oxidized forms of OxyR during normal, non-stressed growth. While the hypothesis that PgdX acts as a negative controller is novel in the case of *H. influenzae*, glutaredoxins in other organisms have been implicated in the relaxation of the OxyR response. For example, in *E. coli* the OxyR regulated glutathione reductase (*gorA*) and glutaredoxin (*grxA*) have been shown to reduce OxyR° to its OxyR^r^ form [39].

While Harrison et al [24] reported that several genes involved in iron and or heme uptake by *H. influenzae* were upregulated following peroxide treatment, our studies failed to demonstrate similar effects. The window of peroxide exposure in this study was smaller than that used in the previous study (i.e. 4 minutes in the current study and 10 minutes in the previous study). Thus, it is likely that the difference in response of the iron/heme uptake genes reflects the ongoing consequences of oxidative stress and cell damage rather than an immediate response to the stress [24].

The contribution of OxyR to virulence and fitness in appropriate animal models was also examined. In a paired comparison of 10810 and its isogenic OxyR mutant in 5-day old infant rats there was no impact of the mutation on virulence. Mutations in specific members of the OxyR regulon in Hibhave also been shown to have no impact on virulence in the infant rat, including both an *hktE* single knockout and an *hktE/pgdX* double knockout o [29], [31]. Although the *oxyR* mutation did not impact virulence in the infant rat, the mutant was significantly impacted in a mixed infection in the same model. In contrast, in 30-day old weanlings both virulence and fitness of the *oxyR* mutant were significantly impacted. It is likely that the difference in the impact of the *oxyR* mutation on virulence in 5-day old and 30-day old rats reflects the development of the innate immune response in rats. It would be of interest to reassess the impact of mutations in *hktE* in 30-day old rats to determine if a similar differential result occurs.

In a model of OM the *oxyR* mutation had no impact in a virulence study but was significantly impaired in a mixed infection experiment. Thus OxyR has an intrinsic contribution to survival in this environment.

Mutants in *pgdX* were also examined in both the chinchilla ear and the two rat models. None of the models showed a difference between the wild-type isolate and the *pgdX* mutant, however all models showed a clear difference in the fitness of the mutant isolate. Other workers have shown a potential contribution of PgdX to virulence in the lung, and have shown expression of PgdX in pooled human sputum [40], [41].

The observation that the *pgdX* mutant exhibited constitutive expression of members of the OxyR regulon may explain the decrease in fitness of this mutant *in vivo*, since in this isolate there would be a higher metabolic demand for energy, possibly increased metal ion uptake via YfeABCD and potentially perturbed redox balance.

Since the response of the OxyR regulon has only been determined *in vitro*, the expression of the regulated genes *in vivo*, during infection of the chinchilla ear, was examined. Catalase, the Yfe metal uptake locus and the 6-phosphogluconate-dehydrogenase were all upregulated in this environ while the NAD(P)H peroxide was un-induced. The peroxiredoxin and Dps protein showed variable regulation. In ears inoculated with the OxyR mutant *pgdX*, *hktE* and *dps* were no longer transcribed at an increased level, while *yfeA* and *gnd* remained induced. The reasons behind this lack of induction of several of the OxyR members in the chinchilla ear are at present unknown, but most likely represent differential regulation of these genes in this environment. For example, it is known that *dps* transcription is also regulated by ArcA [32] and similarly the *yfeABCD* operon is under the control of iron and/or heme levels [11], [12].

In summary, we have demonstrated that the *oxyR* mediated response of *H. influenzae* to oxidative stress is a very rapid, tightly controlled event, possibly controlled by both a negative feedback loop via the action of induced PgdX on OxyR and by autoregulation of *oxyR* transcripts. Transcripts of regulon members are upregulated during OM, although. mutants lacking the *oxyR* gene are impacted only in fitness and not in virulence.

## Methods

### Bacterial Strains and Growth Conditions

NTHi strain 86-028NP is a nasopharyngeal isolate from a patient who underwent tympanostomy and tube insertion for chronic otitis media, and has been extensively described in chinchilla models of otitis media [42]–[45]. The *H. influenzae* type b (Hib) strain 10810 was isolated from a case of meningitis and its genome has been completely sequenced (GenBank Accession No. NC_016809). The sequenced *H. influenzae* strain Rd KW20 was obtained from the ATCC [46]. Isolates of *H. influenzae* were routinely maintained on chocolate agar with bacitracin at 37°C. When necessary *H. influenzae* were grown on brain heart infusion (BHI) agar supplemented with 10 µg/ml heme and 10 µg/ml β-NAD (supplemented BHI; sBHI) and the appropriate antibiotic(s). *Escherichia coli* TOP10 (Invitrogen) was used for cloning experiments and was routinely grown on LB agar supplemented with the appropriate antibiotics. Spectinomycin was used at 200 µg/ml in *H. influenzae* and 100 µg/ml in *E. coli,* chloramphenicol was used at 3 µg/ml in *H. influenzae* and 100 µg/ml in *E. coli.*

### Construction of *oxyR* and *pgdX* Deletion Mutants

Mutants were essentially constructed as described previously [4], [8]. For deletion of *oxyR*, two pairs of primers were designed to amplify regions upstream and downstream of *oxyR* respectively. Primer pair HI0571UPF and HI0571UPR was used in a PCR to amplify a ∼1,000-bp product upstream of *oxyR* with engineered *Eco*RI and *Bam*HI sites at the ends of the amplicon to allow for directional cloning. The second primer pair, HI0571DNF and HI0571DNR, was designed to amplify an ∼1,000-bp product downstream of *oxyR* in the PCR and similarly add *Hind*III and *Bam*HI sites to the ends of the amplicon. PCR products of the correct size were cloned into pCR2.1-TOPO and sequentially subcloned into pUC19N using the engineered restriction sites to yield a plasmid that contained the contiguous upstream and downstream sequences of *oxyR* abutting each other with a unique engineered *BamHI* site between them. This construct was digested with *Bam*HI and ligated to a ∼1200-bp *Bam*HI-excised spectinomycin resistance-encoding marker from pSPECR [47] to yield pJS292.

For deletion of *pgdX* two primers targeting the upstream region (PGDUPF and PGDUPR) added *Eco*RI and *Bam*HI sites to the end of the amplicon and two primers targeting the downstream region of *pgdX* (PGDDNF and PGDNR) added *Bam*HI and *Hind*III sites to the end of that amplicon. The two amplicons were sequentially cloned into pUC19N and the spectinomycin-resistance cassette inserted at the unique *Bam*HI site to yield plasmid pJB17.

Competent *H. influenzae* were transformed to spectinomycin resistance with each mutagenic construct, using the static aerobic method as previously described [43], and selected on sBHI agar containing spectinomycin. Correct chromosomal recombinations were confirmed by the molecular size of a PCR product resolved on an agarose gel (data not shown). Primers used in the PCRs are listed in Table 2.

**Table 2:** Primer sequences used in this study.

Primer	Target	Sequence		
HI0571UPF	*oxyR*	GAATTCTGCCTGAACTTTCGCGTAATAAC		
HI0571UPR	*oxyR*	GGATCCTTGAAATAATGGCTGGTGTTCGT		
HI0571DNF	*oxyR*	GGATCCAGATTTTCCCTATAGAAAAATTC		
HI0571DNR	*oxyR*	TCTTCAAAGCTTAAGCCTTTATCGTGTA		
PGDUPF	*pgdX*	GAATTCAGGACGATAAACCAACG		
PGDUPR	*pgdX*	GGATCCTGTTTTTTCTCCTATTGG		
PGDDNF	*pgdX*	GGATCCTTTAGCTAATTATTATTTGGG		
PGDDNR	*pgdX*	AAGCTTATCAAATACTTTTCCATTTGG		
OXYR-COMPF	*oxyR*	GGATCCACTAGACATTGTTTTTTCTCCTATTG		
OXYR-COMPR	*oxyR*	GGATCCAAATTAGAAAACTTTTTTCTGCGC		
QPCR-HI1264-uF	*gyrA*	CGTAAAATCAGCGCGTGTTG		
QPCR-HI1264-R2	*gyrA*	GAGAATGGTTGTGCCATACGA		
QPCR-HI0361-uF	*yfeB*	GGCAAGTCCACATTATTTAAAAGCAT		
QPCR-HI0361-uR	*yfeB*	TGGGAAATTGGCAAATCACAA		
QPCR-HI0553-uF	*gnd*	CGAAGTGGGTATCCCAATGC		
QPCR-HI0553-uR	*gnd*	AAACGTGCTGAAGTATAGCCATCTAAG		
QPCR-HI0571-uF	*oxyR*	GGGCCATTACACATCGGTTT		
QPCR-HI0571-uR	*oxyR*	CCGCTTTTAACATTGGCACAA		
QPCR-HI0572-uF	*pgdX*	AACCAGGCGATCCGTTCA		
QPCR-HI0572-uR	*pgdX*	TTGGTGTTGTGGTGCAAGGT		
QPCR-HI0928-uF	*hktE*	TTGAAGTCGGCGAGTTTGAA		
QPCR-HI0928-R1	*hktE*	TGCAAATGCAGATTGTTCTACA		
QPCR-HI1349-uF	*dpsA*	TGTCAAAAACATCAATCGGACTAGA		
QPCR-HI1349-uR	*dpsA*	GGTAGGTTGCAAGTAATTCGTTAAGTT		
QPCR-HI1362-uF	*pntA*	AACGCCGAAAACGGTTCA		
QPCR-HI1362-uR	*pntA*	TTAAAACCTGCATCGTGTTCCA		

### Complementation of the *oxyR* Insertion Mutation

The *oxyR* gene together with approximately 100-bp upstream and 75-bp downstream was amplified by PCR using primers oxyRcompF and oxyRcompR which incorporate *Bam*HI sites at each end. The resulting product was ligated into pCR2.1-TOPO to yield pHJM2 and the fragment confirmed by automated sequencing. pHJM2 was digested with *Bam*HI and the *oxyR* containing fragment was ligated into *Bam*HI digested and dephosphorylated pASK5 to yield pHJM2′. The use of pASK5 allows for complementation of gene disruptions in *H. influenzae* by insertion of a gene in the nonessential outer membrane protein OmpP1 locus [48]. The plasmid pHJM2′ was transformed into *H. influenzae* made competent using MIV media as described by Poje and Redfield [49] and selected on sBHI containing chloramphenicol. Chloramphenicol resistant colonies were identified and the correct chromosomal rearrangements were confirmed by the molecular size of a PCR product resolved on an agarose gel (data not shown). Primers used in the PCRs are listed in Table 2.

### Growth Studies

Growth studies were performed using the Bioscreen C Microbiology Reader (Oy Growth Curves AB Ltd., Helsinki, Finland) as previously described [5], [50]. Growth media were supplemented with graded concentrations of oxidizing agents as detailed in the text. Five replicates of each condition were performed for each experiment, and each experiment repeated at least twice.

### Growth of Bacteria for Q-PCR Analysis

To assess gene expression under conditions of oxidative stress, growth experiments were performed and RNA profiles established before and after addition of perturbant. To prepare the primary inocula, *H. influenzae* were initially grown in 15 ml conical tubes containing 5 ml of BHI broth supplemented with 10 µg/ml β-NAD and 2.0 µg/ml heme at 37°C on a rotator for 2 hours. For the final inoculum, cells were pelleted by centrifugation, washed once in phosphate buffered saline (PBS) containing 0.1% gelatin and the pelleted cells were re-suspended in the same buffer. The suspension was adjusted to an A_605nm_ = 0.50 and then diluted serially in the same buffer to provide an inoculum giving a final concentration of ∼2×10^8^ cfu/ml when 5 ml of inoculum was added to 60 ml sBHI. Broth cultures were incubated in a rotary shaker at 175 rpm at 37°C and samples taken for viable counts (50 µl) and RNA extraction (500 µl) at 30 minute intervals. Immediately following the 60 minutes sampling, H_2_O_2_ was added to each test flask to give a final concentration of 150 µM and 500 µl samples were removed at 2 minute intervals for RNA extraction. Further samples for viable counts were removed every 30 minutes. To ensure preservation of the RNA profiles, each 500 µl sample was immediately mixed upon extraction with 1 ml RNAProtect (Qiagen, Valencia, CA) and frozen at −70°C for later RNA preparation.

### RNA Purification

*In vitro* samples for Q-PCR obtained as described above were thawed, remixed by brief vortexing and incubated at room temperature for 5 minutes prior to purification using the RNeasy mini kit (Qiagen, Valencia, CA). Following purification, the sample was eluted with 40 µl of sterile RNase free water. Residual chromosomal DNA was removed by digestion with TurboDNase (Ambion, Applied Biosystems, Foster City, Ca). The RNA samples were used to prepare cDNA as previously described [51]. Each 20 µl cDNA reaction contained 7 µl template RNA, 5.5 mM MgCl_2_, 500 µM each dNTP (dATP, dCTP, dGTP, dTTP), 1x RT buffer, 80 mU RNase Inhibitor and 25 U MultiScribe Reverse Transcriptase (Applied Biosystems, Foster City, Ca.). The synthesis reaction was incubated at 25°C for 10 minutes followed by a further 30 minutes at 48°C. The reaction was terminated by heating at 95°C for 5 minutes. Prior to analysis, the cDNA was diluted by addition of 180 µl RNase-free water.

### Quantitative Real-time PCR

Q-PCR was performed as previously described [51]. Gene-specific oligonucleotide primers were designed using Primer Express 2.0 (Applied Biosystems), synthesized by Operon Technologies (Huntsville, AL) and were tested to determine amplification specificity, efficiency and for linearity of the amplification with RNA concentration (For primer sequences see Table 2). A typical 10 µl reaction contained 5 µl of SYBR Green Master Mix, 10 nM of each primer, and 2.5 µl of cDNA sample. Quantification reactions for the target transcripts at each timepoint were performed in quadruplicate and normalized to concurrently run *gyrA* from the same sample. Relative quantification of gene expression was determined using the 2^−ΔΔCt^ method of Livak and Schmittgen where 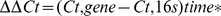 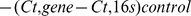 [52].

### Ethics Statement

This study was performed in strict accordance with the recommendations in the Guide for the Care and Use of Laboratory Animals of the National Institutes of Health. The protocols for use of animals in this study were reviewed and approved by the Institutional Animal Care and Use Committee of the University of Oklahoma Health Sciences Center (Protocol numbers 11-031I and 11-033I). All procedures on rats were carried out under anesthesia with gaseous isoflurane. All procedures on chinchillas were carried out under anesthesia with ketamine (10 mg/kg) and xylazine (2 mg/kg). All animals were monitored daily as described in the specific animal model sections. Rats that were hypothermic or exhibited other signs of severe infection determined in consultation with the veterinary staff of the animal facility were killed by CO_2_ inhalation. Chinchillas exhibiting signs of inner ear and/or systemic infection were killed by CO_2_ inhalation. All animals were killed upon completion of the experimental protocol by CO_2_ inhalation.

### Rat Models of Invasive Disease

Rat models of bacteremia following intraperitoneal infection with *H. influenzae* were used in 8 separate experiments to compare the abilities of strains to produce invasive disease as previously described [4], [28].

Specified pathogen free (SPF), timed-pregnant Sprague-Dawley rats (Harlan Sprague-Dawley) were received approximately five days prior to giving birth. These pregnant females were single housed on hardwood litter with *ad libitum* access to water and a standard pelleted food (Purina Lab Rodent Diet 5001). They were maintained on a 12 hour light-dark cycle in separate forced air cubicles in a bio-containment facility to prevent cross-contamination. Newborn pups from different mothers were pooled and randomly reassigned to the mothers (n = 10 pups per female).

In the first experiment groups of ten 5-day old infant rats were inoculated by intraperitoneal injection with approximately 100 CFU of either the Hib strain 10810 or its *oxyR* mutant derivative HI2283 in order to compare virulence of the two strains. Inocula were prepared as previously described [4]. The actual infective dosage was confirmed by triplicate plating 100 µl aliquots of the inocula on chocolate agar containing bacitracin. At 24 hour intervals pups were examined for signs of infection (neurological symptoms: tremor, loss of righting ability, coma, rigidity; systemic symptoms: immobility/lethargy; anorexia, poor grooming/ruffled fur, hypothermia). Blood specimens (50 µl) were obtained from anesthetized animals (gaseous isoflourane) by cardiac puncture. Bacterial titers were determined using a modification of the track-dilution method of Jett et al as previously described [4], [53]. Serial dilutions (0 to 10^−5^) of freshly drawn whole-blood were made in 0.1% gelatin in PBS and 10 µl aliquots from each dilution were plated in triplicate on sBHI and all plates were incubated at 37°C for 24–48 hours to quantify CFU NTHi/ml.

In the second experiment a group of ten 5-day old infant rats was inoculated by intraperitoneal injection with a mixture containing equal numbers of both the Hib strain 10810 or its *oxyR* mutant derivative HI2283 (total of approximately 200 CFU equally divided between the strains) in order to assess differential fitness of the two strains. Infected rats were assessed as described above. Each freshly drawn whole-blood sample was plated on sBHI and on sBHI containing 200 µg ml ^−1^ spectinomycin to determine total bacterial titer and the titer of the mutant strain respectively.

Experiments 3 and 4 were identical to experiments 1 and 2 except that they compared strain 10810 to the isogenic *oxyR* mutant strain HI2283 in 30-day old weanling rats with infective doses of approximately 2,000 CFU in both experiments (in the mixed infection the 2,000 CFU was equally divided between the two strains).

Experiments 5–8 were identical to experiments 1–4 except that they compared strain 10810 to its isogenic *pgdX* mutant HI2159.

### Chinchilla Model of Otitis Media

A total of 32 adult chinchillas (*Chinchilla lanigera*) with no evidence of middle ear infection by either otoscopy or tympanometry at the beginning of the study were used. Animals were rested for at least 7 days upon arrival to acclimate them to the vivarium. Chinchillas were single housed on corn cob litter with *ad libitum* access to water and a standard pelleted food. After acclimation, chinchillas were challenged with *H. influenzae* in five separate experiments as previously described [43], [54]. Animals were monitored daily for signs of inner ear infection and/or systemic infection including head tilt and loss of righting ability. Animal procedures have been described in detail elsewhere [44], [55], [56].

In the first experiment groups of five chinchillas were challenged in both ears transbullarly with approximately 500–1000 CFU of either NTHi strain 86-028NP or its *oxyR* mutant derivative HI2285 in order to compare virulence of the two strains. Transbullar inocula were delivered in 300 µl 0.1% gelatin in PBS by direct injection of bacterial suspensions into the superior bullae. Actual challenge dosages received were confirmed by plate count. On days 4, 7, 11, 14 18, 22 and 28 post challenge MEE were collected by epitympanic tap, i.e. withdrawl of fluids from the middle ear cavity using a 1.5 inch 25-gauge hypodermic needle [56]. Ears were scored as “dry” (i.e. no detectable MEE) when an ear was successfully tapped and no evidence of effusion was seen when the plunger of a 1 ml syringe was pulled back maximally. In some cases ears were scored as “ISVP” (insufficient volume to plate) when there was any evidence of effusion, which in some cases manifested as bubbles in the hub of the syringe, but the volume was insufficient to perform a dilution series.

Bacterial titers were determined using the track dilution method. Serial dilutions (0 to 10^−5^) of freshly recovered MEE were made in 0.1% gelatin in PBS and 10 µl aliquots from each dilution were plated in triplicate on sBHI and all plates were incubated at 37°C for 24–48 hours to quantify CFU NTHi/ml.

In the second experiment a group of 5 chinchillas was challenged transbullarly with an inoculum containing equal numbers of NTHi strain 86-028NP and its *oxyR* mutant derivative HI2285 (total of approximately 1500 CFU) to quantify differential fitness of the two strains. All ears were tapped for collection of MEE on days 4, 7 and 11 days post infection. Each recovered MEE was plated on sBHI and on sBHI containing 200 µg/ml spectinomycin in order to determine total bacterial titer and the titer of the mutant strain respectively.

Experiments 3 and 4 were identical to experiments 1 and 2 except that they compared strain 86-028NP to the isogenic *pgdX* mutant strain HI2334 and that experiment 3 was sampled only through day 18 since in this experiment most ears were dry by this time point.

In the fifth experiment 2 chinchillas were infected transbullarly in both ears with approximately 2,000 CFU of strain 86-028NP. MEE were collected from each ear on days 7, 10, 14 and 17 post infection. Immediately on collection, the samples were mixed with an equal volume of RNAProtect to preserve the RNA profile, prior to purification and analysis by Q-PCR.

### Purification of RNA from Chinchilla Ear Effusions

RNA was isolated from *in vivo* samples as follows: 1 ml Trizol was added to 50 µl of a recovered chinchilla MEE:RNA protect sample collected as described above. The samples were allowed to incubate for 10 minutes at room temperature. To this, 0.4 ml of chloroform was added and the samples briefly mixed. The samples were transferred to a Phaselock Heavy tube (5Prime, Gaithersburg, MD) and the phases separated by centrifugation at 14,000 RPM for 10 minutes at room temperature. The aqueous layer was removed and transferred to another Phaselock Heavy tube and an equal volume of tris-saturated phenol-chloroform (pH 6.7) was added and the solution briefly mixed. The phases were separated by centrifugation at 14,000 RPM for 10 minutes at room temperature. The aqueous phase was transferred to a new tube and the RNA was precipitated with 0.1×volume 3 M sodium acetate, 0.6×volume isopropanol, and 45 µg Glycoblue (Life Technologies). After incubation at −20°C overnight, the RNA was pelleted by centrifugation at 14,000 RPM for 15 minutes at room temperature. Residual salt was removed by washing the pellet once in 75% ethanol. The pellets were resuspended in 50 µl of RNase free water. Residual DNA was removed by treartment with TurboDNase. Prior to Q-PCR, the cDNA was prepared in the same manner as performed with *in vitro* samples.

### Statistics

Statistical comparisons of growth between strains under the same growth conditions in vitro were made using the Mann-Whitney test.

For in vivo studies bacterial titers in blood or MEE are expressed as mean±SD typically from groups of 10 rats or in the case of chinchilla experiments 10 ears. Bacterial titers were compared using the Mann-Whitney test.

Percentages of infected rats and percentages of MEE with detectable bacteria were compared using the Fisher Exact Test.

Mann-Whitney tests were performed using Analyze-It for Microsoft Excel v2.22 (Analyze-It Software Inc., Leeds, England). Fisher Exact tests were performed with GraphPad (http://www.graphpad.com/quickcalcs/contingency1.cfm). A P value<0.05 was taken as statistically significant for all analyses.
